# Molecular Docking, Metabolite Profile, In Vitro Antioxidant, Anticholinergic, Antidiabetic, and Antiglaucoma Properties of Royal Jelly

**DOI:** 10.3390/metabo16100729

**Published:** 2026-09-29

**Authors:** Ebubekir İzol, Ülkü Yerebasan, Esma Kayaoğlu, Münire Turhan, Mustafa Abdullah Yılmaz, Adem Necip, Rüya Sağlamtaş, Hülya Akıncıoğlu, İlhami Gülçin

**Affiliations:** 1 Department of Bee and Bee Products, Institute of Science, Bingöl University, 12000 Bingöl, Türkiye; 2 Bee and Natural Products R&D and P&D Application and Research Center, Bingöl University, 12000 Bingöl, Türkiye; 3 Department of Pharmacology and Toxicology, Faculty of Veterinary Medicine, Bingöl University, 12000 Bingöl, Türkiye; uyerebasan@bingol.edu.tr; 4 Department of Food Hygiene and Technology, Faculty of Veterinary Medicine, Bingöl University, 12000 Bingöl, Türkiye; ekayaoglu@bingol.edu.tr; 5 Institute of Health Sciences, Ankara University, 06100 Ankara, Türkiye; 6 Faculty of Agriculture, Vocational School of Food, Agriculture and Livestock, Bingöl University, 12000 Bingöl, Türkiye; mturhan@bingol.edu.tr; 7 Department of Pharmaceutical Chemistry, Faculty of Pharmacy, Dicle University, 21280 Diyarbakır, Türkiye; mustafaabdullahyilmaz@gmail.com; 8 Department of Pharmacy Services, Vocational School of Health Services, Harran University, 63000 Şanlıurfa, Türkiye; ademnecip@harran.edu.tr; 9 Medical Services and Techniques Department, Vocational School of Health Services, Ağrı İbrahim Çeçen University, 04100 Ağrı, Türkiye; rsaglamtas@agri.edu.tr; 10 Department of Biochemistry, Faculty of Pharmacy, Ağrı İbrahim Çeçen University, 04100 Ağrı, Türkiye; hgakincioglu@agri.edu.tr; 11 Ağrı İbrahim Çeçen University Rectorate, 04100 Ağrı, Türkiye; igulcin@atauni.edu.tr; 12 Department of Chemistry, Faculty of Science, Ataturk University, 25240 Erzurum, Türkiye

**Keywords:** royal jelly, antioxidant, metabolite profile, enzyme inhibitions, molecular docking

## Abstract

**Background/Objectives**: Royal jelly is a biologically active bee product with a complex chemical composition and diverse biological properties. This study aimed to comprehensively characterize the phytochemical composition of royal jelly and evaluate its antioxidant capacity, enzyme-inhibitory abilities, and potential molecular interactions with target enzymes. **Methods**: The phytochemical profile of royal jelly was characterized using liquid chromatography–tandem mass spectrometry (LC–MS/MS). Total phenolic and flavonoid contents were determined spectrophotometrically. Antioxidant activity was evaluated using DPPH^•^, ABTS^•+^, and DMPD^•+^ scavenging assays, together with Fe^3+^, CUPRAC, and FRAP reducing methods. Enzyme-inhibitory abilities were assessed against human carbonic anhydrase I and II (hCA I and hCA II), acetylcholinesterase (AChE), butyrylcholinesterase (BChE), α-glucosidase, and α-amylase. Molecular docking analyses were subsequently performed to investigate the interactions of major identified compounds with enzyme active sites. **Results**: LC–MS/MS analysis revealed a diverse profile of phenolic and flavonoids, including quinic acid, caffeic acid, chlorogenic acid, *p*-coumaric acid, apigenin, luteolin, kaempferol, and amentoflavone. Royal jelly exhibited considerable antioxidant activity, with IC_50_ values of 29.33, 29.20, and 21.65 μg/mL in the DPPH^•^, ABTS^•+^, and DMPD^•+^ scavenging assays, respectively. It also demonstrated notable reducing capacity in Fe^3+^ reduction (0.702 μg/mL), CUPRAC (1.444 μg/mL), and FRAP (0.964 μg/mL) assays. Significant inhibitory ability was observed against hCA I (1.12 μg/mL), hCA II (1.33 μg/mL), AChE (1.44 μg/mL), BChE (1.52 μg/mL), α-glucosidase (2.04 μg/mL), and α-amylase (2.11 μg/mL). Docking analyses indicated that major constituents, particularly quinic acid, could interact with key catalytic residues through hydrogen bonding and electrostatic interactions. **Conclusions**: Royal jelly possesses a rich polyphenolic profile accompanied by substantial antioxidant and multi-target enzyme-inhibitory abilities. These findings provide biochemical and molecular evidence supporting the potential of royal jelly as a functional food and nutraceutical ingredient and highlight its value as a source of bioactive compounds for further pharmacological investigation.

## 1. Introduction

Royal jelly (RJ) is one of the most delicate bee products and an important bee product with unique bioactive components. It is produced by the salivary glands of worker bees aged five to fifteen days. It is the primary food source for queen bees and young bees. It has a white-cream color and a viscous texture. Since it spoils quickly, it must be stored in a cold chain [1]. It is used in traditional medicine, and studies have reported its antioxidant, anticancer, antibacterial, anti-inflammatory, anti-wrinkle, and fertility properties. It is consumed as an important food and folk medicine with high protein value [2,3,4,5].

Antioxidants play a crucial role in disease prevention and treatment by scavenging reactive oxygen species without generating secondary radicals [6,7,8,9,10,11]. Among bioactive compounds, secondary metabolites represent a major class responsible for antioxidant activity, with phenolic compounds being particularly prominent [12,13,14,15,16,17].

Extensive research has been conducted on the chemical composition and antioxidant properties of various bee products, including bee venom, propolis, pollen, honey, and apilarnil [18,19,20]. However, the biochemical profile and antioxidant potential of royal jelly remain comparatively less explored, despite its well-documented nutritional and functional significance. Moreover, it has been demonstrated that the chemical composition and biological activities of bee products are strongly influenced by geographical origin, climatic conditions, botanical diversity, and honeybee species.

Although royal jelly is widely recognized for its health-promoting properties, comprehensive biochemical and pharmacological data regarding its enzyme-inhibitory abilities remain limited. Studies investigating the in vitro antioxidant, anticholinesterase, carbonic anhydrase, and carbohydrate-hydrolyzing enzyme-inhibitory abilities of royal jelly remain relatively limited. In particular, the inhibitory effects of royal jelly on acetylcholinesterase and butyrylcholinesterase, enzymes associated with cholinergic neurotransmission; carbonic anhydrase I and II, enzymes involved in physiological carbon dioxide–bicarbonate homeostasis; and α-glucosidase and α-amylase, key enzymes involved in carbohydrate digestion and glucose metabolism, have not yet been comprehensively characterized. These enzyme targets are biochemically relevant to processes associated with neurological function, carbonic anhydrase-mediated physiological regulation, and carbohydrate metabolism, respectively, providing an appropriate basis for evaluating the in vitro biochemical properties of royal jelly without implying clinical or therapeutic efficacy. The lack of integrated data in these areas hampers a full understanding of the therapeutic potential of royal jelly. Therefore, the present study aims to address these gaps and provide a comprehensive evaluation of the antioxidant capacity, chemical composition, and enzyme-inhibitory abilities of royal jelly, thereby contributing valuable insight to the existing literature.

The aim of the present study was to comprehensively characterize the secondary metabolite profile of royal jelly and to systematically evaluate its antioxidant capacity and inhibitory activities against key enzymes, including acetylcholinesterase, butyrylcholinesterase, carbonic anhydrase I and II, α-glucosidase, and α-amylase, complemented by molecular docking analyses to provide mechanistic insights into the potential interactions of selected identified constituents with the investigated enzyme targets. Through this integrated approach, potential molecular-level mechanisms underlying the biological effects of royal jelly will be elucidated, thereby providing a robust scientific basis to support future clinical investigations and applied research involving this functional bee product.

## 2. Material and Methods

### 2.1. Chemicals and Reagents

LC-MS/MS analyses were performed using chemicals and standards necessary for antioxidant and enzyme studies, which were purchased from Sigma-Aldrich (Steinheim, Germany). Acetylcholinesterase (AChE, EC 3.1.1.7, ≥100 U/mg protein, *Electrophorus electricus*), butyrylcholinesterase (BChE, EC 3.1.1.8, *Equus caballus*), α-glucosidase (EC 3.2.1.20, *Saccharomyces cerevisiae*), α-amylase (EC 3.2.1.1, *Sus scrofa*), acetylthiocholine iodide, butyrylthiocholine chloride, p-nitrophenyl-α-D-glucopyranoside (p-NPG), and reference inhibitors (galantamine, acarbose) were purchased from Sigma-Aldrich (Steinheim, Germany). The reagents including DPPH^•^, ABTS^•+^, DMPD^•+^, neocuproine, TPTZ, and antioxidant standards (Trolox, BHT, BHA, α-tocopherol) were obtained from Sigma-Aldrich. LC-grade solvents were supplied by Merck (Darmstadt, Germany). All aqueous solutions were prepared using ultrapure water (18.2 MΩ·cm).

### 2.2. Royal Jelly Sample and Preparation

Royal jelly (RJ) sample was collected during the summer season from the Bingöl Central district of Bingöl Province, Türkiye. The sample was obtained directly from a beekeeper and transported to the laboratory under cold-chain conditions. Upon arrival, the sample was stored at −18 °C in a light-protected container until analysis.

**Figure 1:**
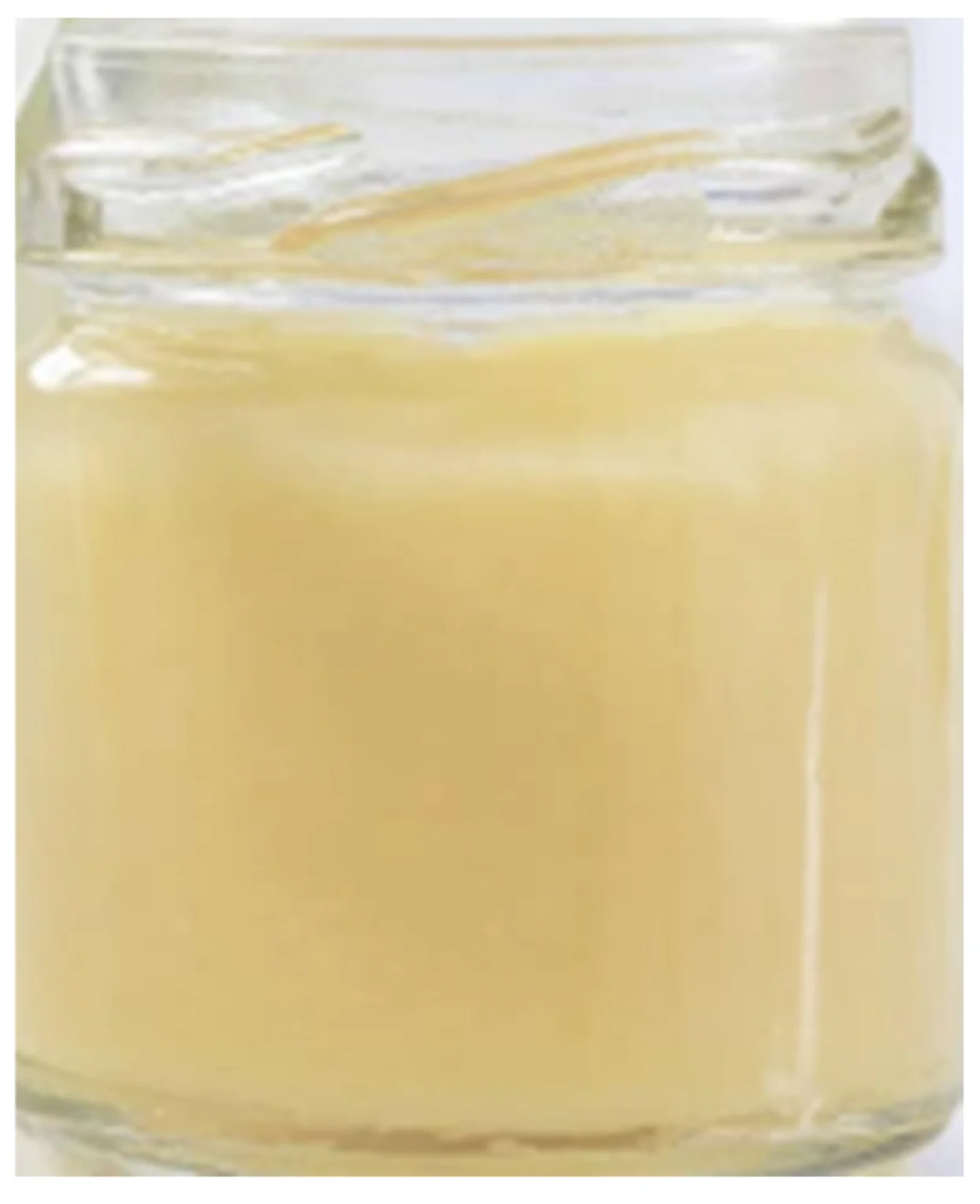
Royal jelly.

For extraction, 3.00 g of RJ (Figure 1) was accurately weighed and transferred to a sealed extraction vessel containing 25 mL of methanol. The mixture was agitated on an orbital shaker at 150 rpm for 24 h at room temperature. Following extraction, the mixture was centrifuged at 3600 rpm for 5 min at 4 °C to precipitate proteins and other insoluble components. The resulting pellet was discarded, and the supernatant was collected and passed through a 0.45 μm membrane filter.

The filtered methanolic extract was concentrated under reduced pressure using a rotary evaporator (BUCHI/R300, Flawil, Switzerland) to remove the solvent. The resulting extract was redissolved in the appropriate solvent at a known concentration and used for subsequent antioxidant and enzyme inhibition assays. This procedure was applied to minimize the presence of residual methanol during the biological assays and to obtain a clarified RJ extract suitable for subsequent analyses [4].

### 2.3. Chemical Identification and Quantification by LC-MS/MS

The chemical composition of the royal jelly extract was analyzed using a Shimadzu LCMS-8040 LC-ESI-MS/MS system (Shimadzu Corporation, Kyoto, Japan) controlled by LabSolutions software ver. 5.53 (Shimadzu). Chromatographic separation was performed using C18 columns (Agilent Poroshell 120 EC-C18, 150 × 2.1 mm, 2.7 μm; or RP-C18 Inertsil ODS-4, 100 × 2.1 mm, 2 μm; Agilent Technologies, Santa Clara, CA, USA). The mobile phases were water containing 5 mM ammonium formate and 0.1% formic acid (A) and methanol containing 5 mM ammonium formate and 0.1% formic acid (B). The gradient was 20–100% B (0–25 min), 100% B (25–35 min), and 20% B (35–45 min). The flow rate and injection volume were 0.5 mL/min and 5 μL, respectively, while the column temperature was maintained at 25–40 °C depending on the analytical conditions.

The MS analysis was performed using electrospray ionization (ESI) (Shimadzu Corporation, Kyoto, Japan). The drying gas (N_2_) flow was 15 L/min, nebulizing gas (N_2_) flow was 3 L/min, DL temperature was 250 °C, heat block temperature was 400 °C, and the interface temperature was 350 °C. Compounds were identified based on their retention times, precursor/product ions, and MS/MS fragmentation patterns by comparison with authentic standards and previously reported data. Quantification was performed using external calibration curves prepared from reference standards, and the results were expressed as μg analyte/g extract. LOD and LOQ values were determined according to the analytical validation procedure applied to the method [21].

### 2.4. Total Phenolic and Flavonoid Contents

Total phenolic content was quantified using the Folin–Ciocalteu reagent, as described in the reference study, with minor adaptations for royal jelly matrix. Briefly, 0.5 mL of extract solution was mixed with 2.5 mL of Folin–Ciocalteu reagent (which diluted 1:10 with distilled water). After standing for 5 min at room temperature, 2.0 mL of 7.5% (*w*/*v*) sodium carbonate solution was added. The mixture was incubated for 60 min in the dark. Absorbance was measured at 760 nm using a UV–Vis spectrophotometer (Thermo Fisher Scientific, Waltham, MA, USA). Results were calculated from a gallic acid calibration curve and expressed as µg gallic acid equivalents (GAE) per g dry weight [22].

Total flavonoid content was determined using the aluminum chloride colorimetric method [15]. Extract (0.5 mL) was mixed with AlCl_3_ (10%, 0.1 mL), potassium acetate (1 M) (0.1 mL), ethanol (4.3 mL), and distilled water. After incubation at 25 °C for 40 min, absorbance was recorded at 415 nm. Results were expressed as µg quercetin equivalents (QE) per g dry weight.

### 2.5. Antioxidant Activity Assay

Following the comprehensive antioxidant evaluation strategy employed in the reference article, multiple assays based on distinct reaction mechanisms were applied.

#### 2.5.1. Ferric Ions (Fe^3+^) Reducing Power Assay

The ferric reducing antioxidant power was determined according to the method of Oyaizu [23], with modifications as described by İzol [10]. For the assay, 67.5, 135, 202.5, or 270 μL of royal jelly extract was mixed with 432.5, 365, 297.5, or 230 μL of distilled water, respectively. Subsequently, 1250 μL of phosphate buffer, 250 μL of FeCl_3_ solution, 1250 μL of K_3_Fe(CN)_6_ solution, and 1250 μL of TCA solution were added. The final volume was 4500 μL. The mixtures were vortexed, and 1 mL of each mixture was transferred to a new tube and mixed with 1 mL of distilled water. After vortexing, the mixtures were incubated for 10 min in the dark at room temperature, and absorbance was measured at 700 nm. Distilled water was used as the blank, while the control was prepared by replacing the sample with distilled water.

#### 2.5.2. Cu^2+^ Reducing Antioxidant Capacity (CUPRAC) Assay

The cupric ion reducing antioxidant capacity (CUPRAC) assay was performed according to the method of Apak et al. [24]. For the assay, 15, 30, 45, or 60 μL of royal jelly extract was mixed with 685, 670, 655, or 640 μL of distilled water, respectively. Then, 125 μL of CuCl_2_ solution, 125 μL of acetate buffer, and 50 μL of neocuproine solution were added to each tube, giving a final volume of 1000 μL. The mixtures were vortexed and incubated for 30 min at room temperature in the dark. Absorbance was measured at 450 nm against distilled water as the blank. The assay was based on the reduction of Cu^2+^ to Cu^+^ by the royal jelly extract.

#### 2.5.3. Ferric Reducing Antioxidant Power (FRAP) Assay

FRAP reagent, consisting of acetate buffer, TPTZ solution, and FeCl_3_·, was freshly prepared and reacted with royal jelly extract. The FRAP assay was performed using the TPTZ method. For the assay, 37.5, 75, 112.5, or 150 μL of sample/standard solution was mixed with 712.5, 675, 637.5, or 600 μL of sodium acetate buffer, respectively. Then, 1000 μL of FeCl_3_ solution and 750 μL of FRAP reagent were added to each tube, giving a final volume of 2500 μL. The mixtures were vortexed, and 1 mL of the upper phase was transferred to a new tube and mixed with 1 mL of distilled water. After vortexing, the mixtures were incubated for 10 min at room temperature in the dark. Absorbance was measured at 593 nm against sodium acetate buffer as the blank [25].

#### 2.5.4. DPPH^•^ Scavenging Activity

The DPPH^•^ scavenging activity was determined according to the method described previously in [26]. A 1 mM DPPH solution was prepared by dissolving 39.43 mg of DPPH in ethanol and making the volume up to 100 mL. The solution was stirred for 16 h before use. The absorbance of the DPPH^•^ control solution was adjusted to 1.00 ± 0.025 before the assay. For the assay, 20, 40, 60, or 80 μL of sample/standard solution was mixed with 730, 710, 690, or 670 μL of ethanol, respectively, followed by 500 μL of 1 mM DPPH^•^ solution. The final reaction volume was 1250 μL. The mixtures were vortexed and incubated for 30 min at room temperature in the dark. Absorbance was measured at 517 nm against ethanol as the blank.

#### 2.5.5. ABTS^•+^ Scavenging Activity

The ABTS^•+^ scavenging activity was determined according to Re et al. [27]. Before use, the absorbance of the ABTS^•+^ solution was adjusted to 1.00 ± 0.025 using a control solution containing ABTS^•+^ and phosphate buffer. For the assay, 6.25, 12.5, 18.75, or 25 μL of sample/standard solution was mixed with 243.75, 237.5, 231.25, or 225 μL of phosphate buffer, respectively, followed by 1000 μL of ABTS^•+^ solution. The final reaction volume was 1250 μL. The mixtures were vortexed and incubated for 30 min at room temperature in the dark, and absorbance was measured at 734 nm. Phosphate buffer was used as the blank.

#### 2.5.6. DMPD^•+^ Scavenging Activity

The study was conducted according to the method of Gulcin [28]. The DMPD^•+^ was prepared by adding 1 mL of 0.1 M DMPD and 0.2 mL of 0.05 M FeCl_3_ to 100 mL of 0.1 M sodium acetate buffer (pH 5.25), yielding a final concentration of 0.01 M DMPD^•+^. Its absorbance was adjusted to 1.00 ± 0.025 before use. For the assay, 22.5, 45, 67.5, or 90 μL of sample/standard solution was mixed with 477.5, 455, 422.5, or 410 μL of distilled water, respectively, followed by 1000 μL of DMPD^•+^ solution. The final volume was 1500 μL. The mixtures were vortexed and incubated for 50 min at room temperature in the dark, and absorbance was measured at 505 nm. Sodium acetate buffer was used as the blank.

### 2.6. Enzyme Inhibition Assays

#### 2.6.1. Acetylcholinesterase and Butyrylcholinesterase Inhibition Assay

Acetylcholinesterase (AChE) and butyrylcholinesterase (BChE) inhibitory activities were determined using the Ellman’s method [29,30]. The assay is based on the hydrolysis of acetylthiocholine or butyrylthiocholine by the corresponding enzyme to produce thiocholine, which reacts with 5,5′-dithiobis-(2-nitrobenzoic acid) (DTNB) to form the yellow-colored 5-thio-2-nitrobenzoic acid, measured at 412 nm. For the assay, each reaction mixture contained 100 μL of phosphate buffer, 780 μL of distilled water, 50 μL of DTNB solution, 20 μL of enzyme solution, and an appropriate volume of the sample extract as inhibitor. The volume of the sample was adjusted according to the tested concentration, and the volume of distilled water was correspondingly adjusted to maintain a final reaction volume of 1000 μL. Following pre-incubation, 50 μL of substrate solution was added to initiate the reaction. Acetylthiocholine iodide was used as the substrate for AChE, whereas butyrylthiocholine iodide was used for BChE. AChE (EC 3.1.1.7, Electrophorus electricus) and BChE (EC 3.1.1.8, *Equus caballus*) were used as the enzyme sources. Tacrine was used as the reference inhibitor for both AChE and BChE assays.

The change in absorbance at 412 nm was monitored for 3 min. Enzyme inhibition was evaluated by calculating the percentage of residual enzyme activity relative to the control. IC_50_ values were determined by plotting the percentage of enzyme activity against inhibitor concentration and calculating the concentration corresponding to 50% inhibition.

#### 2.6.2. α-Amylase Inhibition Assay

The α-amylase inhibitory effect was determined according to the method described by Xiao et al. [31]. Commercially available α-amylase (E.C.3.2.1.1, *Sus scrofa*) was used. The reaction mixture consisted of 500 μL of starch solution, 250 μL of buffer, 250 μL of enzyme solution, and an appropriate volume of royal jelly extract as the inhibitor. The volume of the buffer was adjusted according to the volume of sample added to maintain a final reaction volume of 1000 μL. Starch was dissolved using a heated magnetic stirrer until complete dissolution before use. Different concentrations of the royal jelly extract were tested to determine the inhibitory activity. The reaction was monitored spectrophotometrically at 580 nm, and the percentage of enzyme inhibition was calculated relative to the control. The IC_50_ value was determined by plotting the percentage of residual enzyme activity against the inhibitor concentration. Acarbose was used as the reference inhibitor, and the results were expressed as IC_50_ (μg/mL).

#### 2.6.3. α-Glucosidase Inhibition Assay

The α-glucosidase inhibitory effect was determined according to the method described by Tao et al. [32]. The reaction mixture consisted of 100 μL of buffer, 820 μL of distilled water, 30 μL of α-glucosidase enzyme solution, and 50 μL of *p*-nitrophenyl-α-D-glucopyranoside (*p*-NPG) substrate. An appropriate volume of royal jelly extract was added as the inhibitor, and the volume of distilled water was adjusted accordingly to maintain a final reaction volume of 1000 μL. The α-glucosidase enzyme used was obtained from *Saccharomyces cerevisiae*. Six different concentrations of the royal jelly extract were tested for determination of the IC_50_ value. The absorbance was measured at 405 nm in 3 min, and the percentage of enzyme inhibition was calculated relative to the control. The IC_50_ value was determined by plotting the percentage of residual enzyme activity against the inhibitor concentration. Acarbose was used as the reference inhibitor, and the results were expressed as IC_50_ (μg/mL).

#### 2.6.4. Carbonic Anhydrase Isoenzymes Inhibition Assay

The esterase activities of human carbonic anhydrase I (hCA I) and II (hCA II) were determined using *p*-nitrophenyl acetate (PNPA) as the substrate, according to the method of Verpoorte et al. [33]. The reaction mixture contained 400 μL of Tris-SO_4_ buffer, 220 μL of distilled water, 360 μL of PNPA solution, and 20 μL of enzyme solution, giving a final volume of 1000 μL. For inhibition studies, the volume of distilled water was adjusted according to the volume of royal jelly extract used as the inhibitor. PNPA solution was freshly prepared in acetone and protected from light. Absorbance changes were monitored at 348 nm for 3 min [34].

For IC_50_ determination, six different concentrations of the royal jelly extract prepared in DMSO were tested. The percentage of residual enzyme activity was plotted against inhibitor concentration, and IC_50_ values were calculated from the resulting regression equation. Acetazolamide was used as the reference inhibitor. The inhibition results were expressed as IC_50_ (μg/mL) [35].

### 2.7. Molecular Docking

#### 2.7.1. Protein Preparation

Docking studies were performed using the Schrödinger Suite 2023-3 software package through the Maestro interface (Schrödinger, LLC, New York, NY, USA) (https://www.schrodinger.com (accessed on 28 July 2026)). Prior to molecular docking, protein structures were prepared using the Protein Preparation Wizard module in Schrödinger Maestro. During this process, protein structures were carefully examined, and crystallographic water molecules and unnecessary heteroatoms located outside the binding site were removed. Missing side chains and atoms were automatically completed, hydrogen atoms were added, and the protonation states of amino acid residues were adjusted according to physiological pH conditions (pH 7.4). Finally, the prepared protein structures were subjected to energy minimization using the OPLS4 force field to obtain optimized conformations suitable for docking analyses [36].

#### 2.7.2. Ligand Preparation

The structures of the ligands used in the docking analyses were obtained from the PubChem database. Possible ionization and tautomeric states of the ligands were generated at physiological pH (7.0 ± 0.2) using the Epik tool (Schrödinger Release 2026-3). During this process, stereochemical properties were preserved, ligand geometries were optimized, and the structures were subjected to energy minimization under the OPLS4 force field to obtain stable conformations suitable for docking analyses [37].

#### 2.7.3. Grid Generation

For protein–ligand docking analyses, the binding sites were defined based on the co-crystallized ligands present in the 6B3R structure, and three different chains were evaluated separately. The receptor grids corresponding to the active binding pockets were generated using the Receptor Grid Generation module, and the grid box dimensions were optimized to allow proper accommodation of the ligands within the binding site [38].

#### 2.7.4. Molecular Docking Analysis

The prepared ligands were docked into the predefined grid regions using the Glide module of the Schrödinger suite. Docking calculations were carried out in both Standard Precision (SP) and Extra Precision (XP) modes. Ligand flexibility was allowed during the docking process, while the protein structures were treated as rigid. The docking poses with the lowest binding free energy values (GlideScore) were selected as the most probable ligand–protein binding conformations [39].

#### 2.7.5. Interaction Analysis and Visualization

Docking results were analyzed using the Maestro interface and the Ligand Interaction Diagram tools (Schrödinger Release 2026-3). Hydrogen bonds, electrostatic interactions, hydrophobic contacts, as well as π–π and π–cation interactions formed within the ligand–protein complexes were examined in detail. The best-ranked docking poses were visualized to enable a comparative evaluation of the binding modes.

### 2.8. Statistical Analysis

All experimental measurements were performed in triplicate as technical replicates using the same royal jelly sample. These repeated measurements were performed to assess analytical repeatability and were not considered independent biological replicates. Therefore, the triplicate measurements were not used to perform inferential statistical comparisons among independent samples or groups. The results are presented as mean ± standard deviation (SD) of the technical replicates and are intended to describe the analytical variability of the measurements. IC_50_ values were calculated by nonlinear regression analysis, and the goodness-of-fit of the corresponding concentration–response curves were evaluated using the coefficient of determination (r^2^). Because the present study was based on a single independent royal jelly sample, no statistical significance testing between independent biological groups was performed.

## 3. Results

### 3.1. Secondary Metabolite Profile by LC-MS/MS

The results of the secondary metabolite profile of royal jelly determined by LC–MS/MS, together with the main analytical parameters, are presented in Table 1. The secondary metabolites identified in royal jelly included quinic acid, 4-OH benzoic acid, *p*-coumaric acid, protocatechuic acid, chlorogenic acid, caffeic acid, naringenin, luteolin, kaempferol, apigenin, and amentoflavone. Among the detected compounds, quinic acid was the most abundant secondary metabolite, with a concentration of 142,868 µg analyte/g extract.

**Figure 2:**
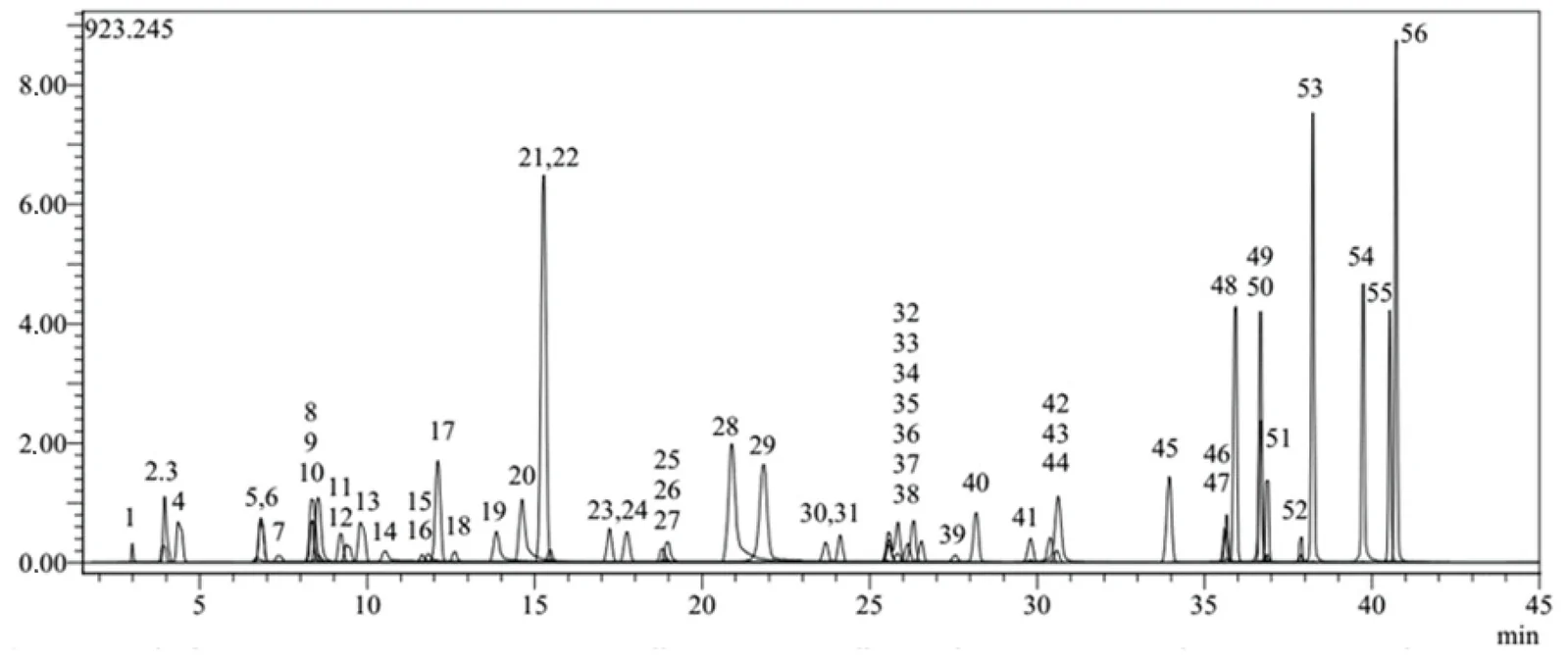
LC-MS/MS chromatogram of reference standard compounds: (1) quinic acid, (2) fumaric acid, (3) aconitic acid, (4) gallic acid, (5) epigallocatechin, (6) protocatechuic acid, (7) catechin, (8) gentisic acid, (9) chlorogenic acid, (10) protocatechuic aldehyde, (11) tannic acid, (12) epigallocatechin gallate, (13) cynarine, (14) 4-hydroxybenzoic acid, (15) epicatechin, (16) vanillic acid, (17) caffeic acid, (18) syringic acid, (19) vanillin, (20) syringaldehyde, (21) daidzin, (22) epicatechin gallate, (23) picein, (24) p-coumaric acid, (25) ferulic acid-d3, (26) ferulic acid, (27) sinapic acid, (28) coumarin, (29) salicylic acid, (30) syringic acid, (31) miquelianin, (32) rutin-d3, (33) rutin, (34) isoquercitrin, (35) hesperidin, (36) o-coumaric acid, (37) genistin, (38) rosmarinic acid, (39) ellagic acid, (40) cosmosiin, (41) quercitrin, (42) astragalin, (43) nicotiflorin, (44) fisetin, (45) daidzein, (46) quercetin, (47) quercetin, (48) naringenin, (49) hesperetin, (50) luteolin, (51) genistein, (52) kaempferol, (53) apigenin, (54) amentoflavone, (55) chrysin, (56) acacetin.

**Figure 3:**
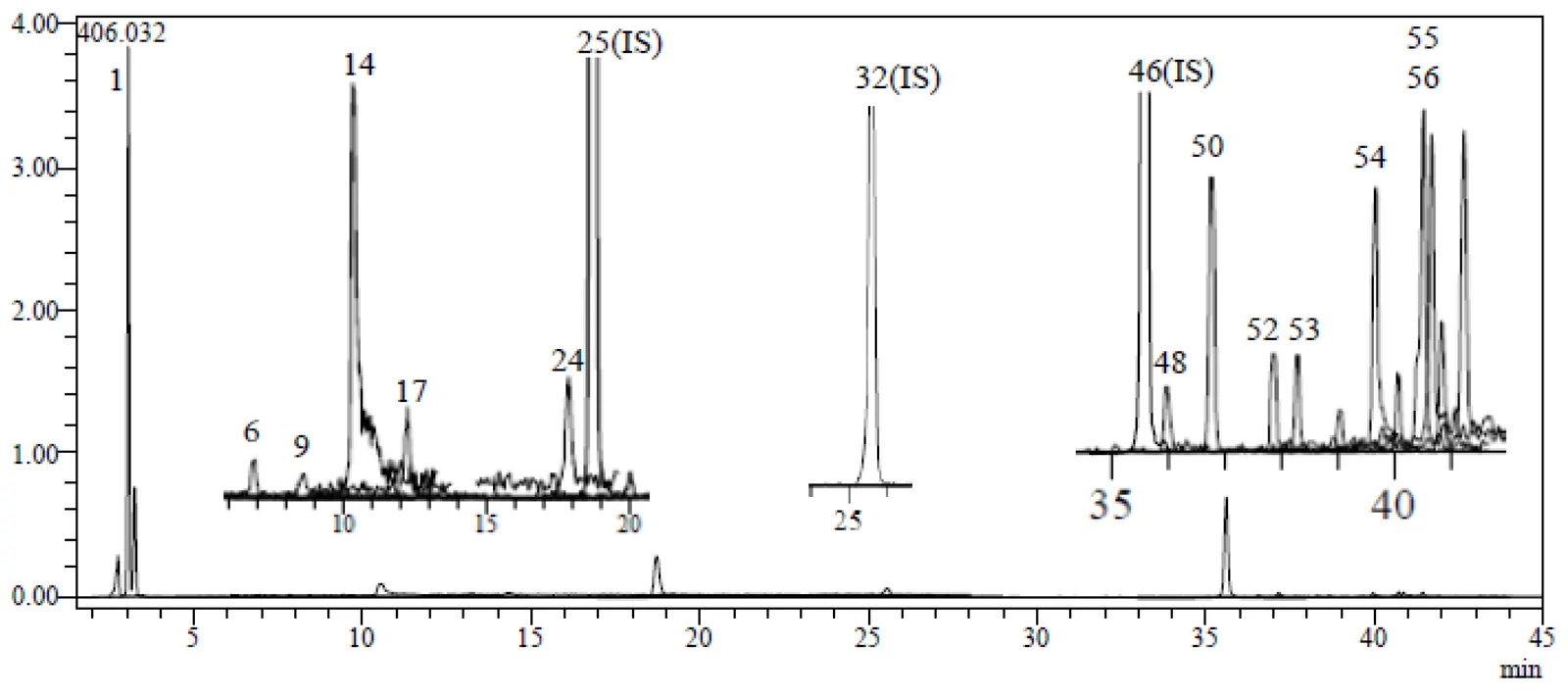
The secondary metabolite chromatogram of royal jelly by LC-MS/MS.

The structures of phytochemicals detected in royal jelly are presented in Table 2. The LC-MS/MS standard chromatogram and the names of the standard secondary metabolites are provided in Figure 2. The secondary metabolite chromatogram of RJ by LC-MS/MS is shown in Figure 3.

### 3.2. Antioxidant Activity Results

The radical scavenging activities of royal jelly were evaluated using DPPH·, ABTS·^+^, and DMPD·^+^ assays, and the results were compared with commonly used synthetic and natural antioxidant standards (Table 3).

Taurine’s indirect antioxidant role against free radicals and its effects on membrane stabilization is important in this context [40].

In the DPPH· scavenging test, royal jelly showed an IC_50_ value of 29.33 ± 2.21 μg/mL. Although this value is higher than the values of five synthetic antioxidants, it is low but close in terms of activity. Nevertheless, it reflects a significant hydrogen donation capacity. This activity can be attributed to the presence of phenolic acids and flavonoids identified in the secondary metabolite profile, including caffeic acid, *p*-coumaric acid, and flavonoids such as apigenin and luteolin, which are known to effectively neutralize stable free radicals.

In the ABTS·^+^ scavenging assay, royal jelly showed an IC_50_ value of 29.20 ± 1.48 μg/mL, comparable to Trolox (27.12 ± 0.85 μg/mL) and ascorbic acid (29.63 ± 2.05 μg/mL). This result suggests that royal jelly possesses a strong electron-transfer capacity, particularly toward hydrophilic radical cations. The relatively strong ABTS·^+^ scavenging activity may be associated with the high abundance of quinic acid and chlorogenic acid, which are known to contribute significantly to total antioxidant capacity in aqueous systems.

Notably, royal jelly exhibited its most pronounced antioxidant activity in the DMPD·^+^ assay, with an IC_50_ value of 21.65 ± 0.96 μg/mL. This value was superior to those of α-tocopherol and ascorbic acid and comparable to Trolox, indicating an effective scavenging ability against nitrogen-centered radicals. The strong performance in the DMPD·^+^ assay suggests that royal jelly may be particularly effective in biological environments where cationic radical species are involved.

Overall, the antioxidant profile of royal jelly across three complementary radical scavenging assays demonstrates that its antioxidant potential is not limited to a single reaction mechanism. Instead, it reflects a multifactorial activity arising from the synergistic effects of phenolic acids, flavonoids, and other bioactive constituents. These findings support the view that royal jelly may serve as a natural antioxidant source with potential protective effects against oxidative stress–related pathological conditions.

In this study, the antioxidant capacity of royal jelly was comprehensively assessed using not only radical scavenging–based assays but also electron transfer–based reducing power methods, including Fe^3+^ reduction, CUPRAC, and FRAP analyses. These complementary approaches provide a more holistic evaluation of antioxidant potential by reflecting different redox mechanisms. The results obtained from the Fe^3+^, CUPRAC, and FRAP reducing power assays for royal jelly, together with those of standard antioxidants, are summarized in Table 4.

In the Fe^3+^ reduction assay, royal jelly demonstrated an absorbance value of 0.702 ± 0.021 at 700 nm, which, although lower than those of strong synthetic antioxidants such as BHA and ascorbic acid, indicates a moderate ferric ion reducing capacity. This activity may be attributed to the presence of phenolic acids, including caffeic acid, chlorogenic acid, and *p*-coumaric acid, which are known to donate electrons and reduce Fe^3+^ to Fe^2+^.

In the CUPRAC assay, royal jelly showed an absorbance of 1.444 ± 0.033 at 450 nm, a value comparable to that of ascorbic acid (1.438 ± 0.022). This finding suggests that royal jelly possesses a substantial cupric ion reducing capacity, particularly in near-physiological pH conditions. The strong performance in the CUPRAC assay is consistent with the high abundance of quinic acid and the diverse flavonoid content identified by LC-MS/MS analysis.

The FRAP analysis revealed that royal jelly exhibited a notable ferric reducing capacity at 593 nm (0.964 ± 0.072; r^2^ = 0.9763). This value is comparable to those of reference antioxidants such as Trolox and ascorbic acid, indicating that royal jelly possesses a strong electron-donating ability. Since the FRAP assay is based on the reduction in the Fe^3+^–TPTZ complex under acidic conditions, these results suggest that royal jelly is particularly effective in electron transfer–based antioxidant mechanisms. This activity is likely attributed to the synergistic effects of phenolic constituents identified by LC-MS/MS analysis, including the high abundance of quinic acid, along with caffeic acid, chlorogenic acid, and flavonoids such as apigenin, luteolin, and kaempferol. Collectively, these findings support the potential of royal jelly as a natural and multifunctional antioxidant source in mitigating oxidative stress–related processes.

In general, the results obtained from Fe^3+^ reduction, CUPRAC, and FRAP assays demonstrate that royal jelly exhibits a robust and multifaceted reducing power profile. The observed antioxidant behavior is likely the result of synergistic interactions among phenolic acids, flavonoids, and other bioactive constituents, supporting the potential of royal jelly as a natural antioxidant source capable of functioning through multiple redox mechanisms.

The total phenolic and total flavonoid contents of royal jelly are presented in Table 5. The results indicate that royal jelly contains appreciable levels of both phenolic compounds and flavonoids. Given that antioxidant activity may be influenced by polyphenolic constituents, the polyphenolic compounds present in royal jelly may contribute to its observed antioxidant potential.

The total phenolic and flavonoid contents of royal jelly determined in this study provide important insight into its antioxidant potential. Royal jelly exhibited a total phenolic content of 53.147 ± 2.224 µg GAE/g and a total flavonoid content of 23.559 ± 1.845 µg QE/g, indicating that it is a meaningful source of bioactive polyphenolic compounds. The levels of polyphenolic compounds detected in royal jelly may contribute to the antioxidant potential observed in the radical scavenging (DPPH, ABTS, and DMPD) and reducing power (FRAP, CUPRAC, and Fe^3+^ reduction) assays. Moreover, the presence of specific phenolic acids and flavonoids identified by LC-MS/MS analysis, such as quinic acid, caffeic acid, chlorogenic acid, apigenin, luteolin, and kaempferol, supports the view that the antioxidant activity of royal jelly arises from a synergistic phytochemical profile rather than from a single dominant compound. These findings reinforce the potential of royal jelly as a natural antioxidant source with possible protective roles against oxidative stress–related pathological conditions.

### 3.3. Enzyme Inhibition Results

In this study, the inhibitory potential of royal jelly against key enzymes involved in metabolic and neurological disorders was comprehensively evaluated across a broad biochemical spectrum. The findings demonstrate that royal jelly exhibits pronounced and multi-target inhibitory activity toward carbonic anhydrase isoenzymes (hCA I and hCA II), as well as enzymes associated with cholinergic function and carbohydrate metabolism. The enzyme inhibition data for royal jelly against hCA I, hCA II, AChE, BChE, α-glucosidase, and α-amylase are summarized in Table 6, expressed as IC_50_ values along with the corresponding coefficients of determination (r^2^).

The enzyme inhibition results demonstrate that royal jelly exhibits pronounced inhibitory activity against multiple clinically relevant enzymes, including hCA I, hCA II, AChE, BChE, α-glucosidase, and α-amylase, with IC_50_ values ranging from 1.12 to 2.11 µg/mL. Notably, royal jelly exhibited strong inhibitory activity against hCA I (IC_50_ = 1.12 µg/mL) and hCA II (IC_50_ = 1.33 µg/mL), with lower IC_50_ values than the reference inhibitor acetazolamide under the experimental conditions employed. These findings demonstrate the pronounced carbonic anhydrase inhibitory activity of the investigated royal jelly sample and support further biochemical investigation of its enzyme-inhibitory properties. Carbonic anhydrase inhibition is an important biochemical property that can be evaluated in the context of enzyme-targeted activity. In the present study, the observed inhibition of hCA I and hCA II demonstrates that the investigated royal jelly sample possesses pronounced carbonic anhydrase inhibitory activity under the experimental conditions employed. However, these findings are limited to the cell-free biochemical assay and should not be interpreted as evidence of ocular, pharmacological, or therapeutic effects.

Royal jelly also demonstrated effective inhibition of cholinesterase enzymes, with IC_50_ values of 1.44 µg/mL for AChE and 1.52 µg/mL for BChE, outperforming the standard drug tacrine. Since cholinesterase inhibitors are central to the management of neurodegenerative disorders such as Alzheimer’s disease [41], the observed cholinesterase inhibitory activity of royal jelly may be of biochemical relevance in the context of cholinergic function. The observed activity may be associated with phenolic acids and flavonoids identified in the secondary metabolite profile, particularly apigenin, luteolin, and *p*-coumaric acid, which have been reported to exhibit anticholinesterase activity or to modulate cholinergic-related pathways [42,43,44].

Furthermore, royal jelly exhibited considerable inhibitory effects against carbohydrate-digesting enzymes, with IC_50_ values of 2.04 µg/mL for α-glucosidase and 2.11 µg/mL for α-amylase, showing greater potency than acarbose. Inhibition of these enzymes is relevant to the biochemical regulation of postprandial glucose metabolism; therefore, the observed inhibitory activity of royal jelly may be of biochemical relevance in the context of carbohydrate-hydrolyzing enzyme activity [45]. Overall, the broad-spectrum enzyme inhibition profile of royal jelly highlights its multifunctional biological activity, likely arising from synergistic interactions among its phenolic compounds, flavonoids, and other bioactive constituents. These results demonstrate that royal jelly exhibits antioxidant and enzyme-inhibitory activities under the experimental conditions employed, providing biochemical evidence of its biological activity.

### 3.4. Molecular Docking

The use of high-resolution crystal structures of target proteins enables a detailed structural evaluation of potential ligand–enzyme interactions. The Protein Data Bank (PDB) is a reliable and widely used repository for structural biology studies of this type. In the present study, crystal structures of enzymes relevant to different biological processes were selected, preferably in complex with known inhibitors, to provide structurally characterized models for molecular docking. The crystal structure of human butyrylcholinesterase (PDB ID: 1P0I, resolution 2.00 Å) was employed as a well-characterized structure relevant to the cholinergic system [46,47,48,49].

Similarly, the crystal structure of recombinant human acetylcholinesterase in complex with galantamine (PDB ID: 4EY6, resolution 2.40 Å) was selected to investigate the potential binding modes of the tested compounds within the cholinesterase binding site. Carbonic anhydrase isoenzymes play important physiological roles, including acid–base regulation and CO_2_ transport, and are recognized as important pharmacological targets [50]. Within this context, the crystal structure of human carbonic anhydrase I in complex with topiramate (PDB ID: 3LXE, resolution 1.90 Å) was selected. The high resolution of these structures provides an appropriate structural basis for evaluating potential ligand–enzyme interactions at the molecular level [51].

Digestive enzymes are also relevant targets in studies of carbohydrate metabolism. The crystal structure of the N-terminal subunit of human maltase–glucoamylase in complex with acarbose (PDB ID: 2QMJ, resolution 1.90 Å) was used as a structural model for evaluating the potential binding of the investigated compounds. Similarly, the crystal structure of human pancreatic α-amylase in complex with myricetin (PDB ID: 4GQR, resolution 1.20 Å) was selected to provide an additional high-resolution model for evaluating ligand–enzyme interactions [52,53].

Molecular docking studies were performed to investigate the predicted interactions of 4-hydroxybenzoic acid, quinic acid, acetazolamide, tacrine, and acarbose with the selected target proteins. The docking results were evaluated using GlideScore values, in which more negative scores generally indicate more favorable predicted ligand–protein interactions within the docking protocol used [54]. However, docking scores represent computational predictions and should not be interpreted as direct measurements or confirmation of experimental inhibitory activity.

The Glide Emodel value was also considered during evaluation of the predicted binding poses. More negative Emodel values may indicate a favorable energetic contribution to the selected docking pose; however, Emodel should likewise be interpreted as a computational scoring parameter rather than a direct measure of binding affinity or biological efficacy. Therefore, docking score and Emodel values were considered together to characterize the predicted binding poses, while the experimental enzyme inhibition results were interpreted independently. The corresponding values are presented in Table 7.

Molecular docking analysis of butyrylcholinesterase (1P0I) showed that quinic acid had the most favorable predicted docking score among the tested ligands (−5.674 Kcal/mol), compared with 4-hydroxybenzoic acid (−4.892 Kcal/mol) and tacrine (−4.236 Kcal/mol). Its relatively favorable Emodel value (−36.535) also supported a favorable predicted binding pose within the modeled binding site. These findings suggest that the multiple hydroxyl and carboxyl groups of quinic acid may facilitate hydrogen-bonding and polar interactions with residues in the enzyme binding region. However, these docking results should be regarded as predictive evidence and do not establish that quinic acid is a more potent BChE inhibitor than tacrine under experimental conditions.

For the maltase–glucoamylase structure (2QMJ), acarbose exhibited the most favorable predicted docking score (−8.020 Kcal/mol) and Emodel value (−106.326) among the tested ligands. Quinic acid showed a more favorable predicted interaction than 4-hydroxybenzoic acid (−6.219 vs. −5.146 Kcal/mol), which may be related to its greater number of hydroxyl groups and corresponding potential for hydrogen-bonding interactions with polar residues in the binding site. Nevertheless, the stronger predicted binding of acarbose is consistent with its highly functionalized structure and extensive capacity for polar interactions. Importantly, the docking result does not confirm the experimental inhibitory activity but provides a structural hypothesis that may help interpret the observed enzyme inhibition.

For carbonic anhydrase I (3LXE), quinic acid (−9.003 Kcal/mol) and 4-hydroxybenzoic acid (−8.447 Kcal/mol) showed more negative predicted docking scores than acetazolamide (−6.296 Kcal/mol). Quinic acid also exhibited a relatively favorable Emodel value (−78.557). The hydroxyl and carboxyl functionalities of these compounds may contribute to hydrogen-bonding and polar interactions within the active-site region. However, the interpretation of docking scores for carbonic anhydrase requires caution because classical inhibitors such as acetazolamide interact directly with the catalytic Zn^2+^ ion, and metal coordination may not be adequately represented by a conventional docking score. Therefore, the present docking results indicate potential binding interactions with CA I but cannot be used to infer superior inhibitory activity relative to acetazolamide [55,56].

For acetylcholinesterase (4EY6), quinic acid exhibited a predicted docking score of −6.662 Kcal/mol, compared with −6.497 Kcal/mol for tacrine and −5.883 Kcal/mol for 4-hydroxybenzoic acid. The favorable predicted binding of quinic acid may be associated with hydrogen-bonding and electrostatic interactions involving its polar functional groups. Nevertheless, the small difference between quinic acid and tacrine in docking score should not be interpreted as evidence of comparable inhibitory potency. Rather, these results suggest that quinic acid can adopt a potentially favorable binding pose within the modeled AChE binding site.

Docking analysis using the second carbohydrate-metabolizing enzyme structure (4GQR) showed that acarbose again had the most favorable predicted docking score (−7.599 Kcal/mol) and a markedly negative E-model value (−105.501). Quinic acid (−6.943 Kcal/mol) and 4-hydroxybenzoic acid (−6.604 Kcal/mol) showed less favorable predicted binding than acarbose. The stronger predicted binding of acarbose may reflect its larger and highly functionalized structure, which allows extensive polar and hydrogen-bonding interactions within the enzyme binding region. The similar ranking observed in the two structural models provides supportive computational evidence for the predicted interaction pattern, but it should not be considered validation or confirmation of experimental inhibition.

In general, the molecular docking analysis suggests that quinic acid can adopt favorable predicted binding poses across several of the investigated enzyme targets, particularly BChE, AChE, and CA I. The structural features of quinic acid, including its multiple hydroxyl groups and carboxyl functionality, may facilitate hydrogen-bonding and other polar interactions with residues within these binding sites. In contrast, acarbose showed the most favorable predicted binding to the maltase–glucoamylase structures, consistent with its highly polar and multifunctional structure. These computational findings provide possible structural explanations for some of the experimentally observed enzyme inhibition results; however, they should be considered hypothesis-generating evidence rather than confirmation of inhibitory activity or therapeutic efficacy. Further kinetic and mechanistic studies are required to establish the contribution of the individual compounds to the observed enzyme inhibition.

**Figure 4:**
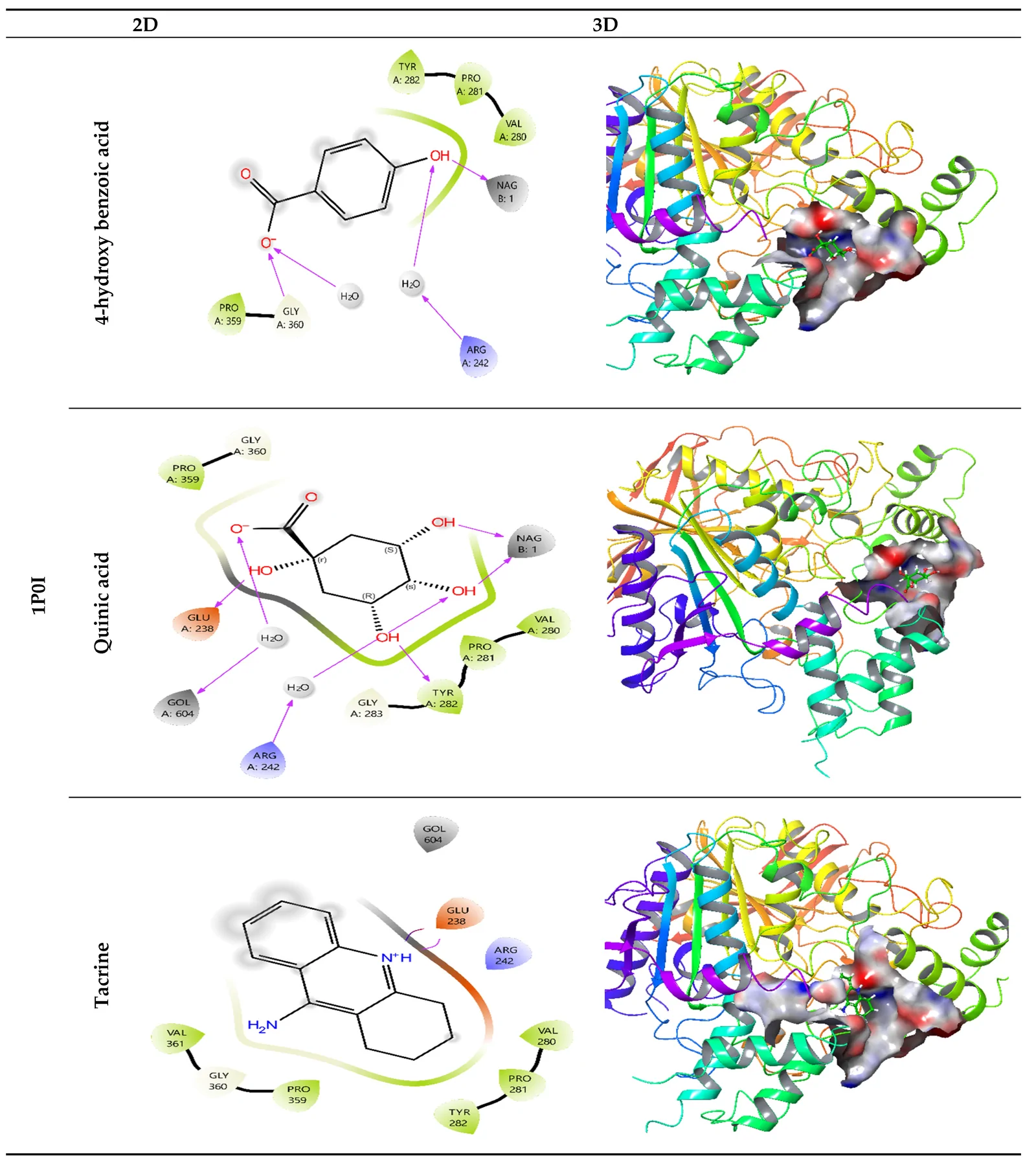

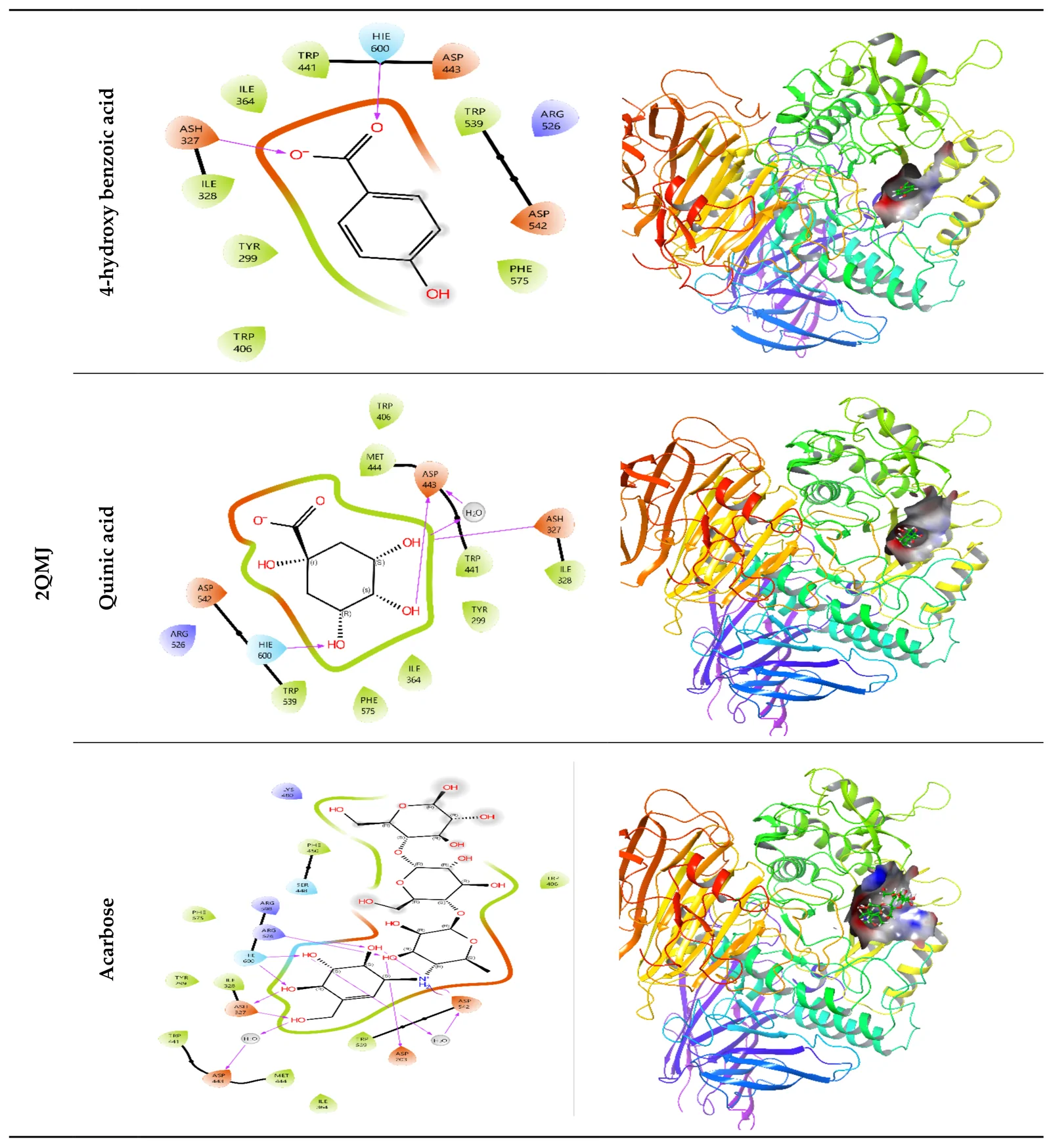

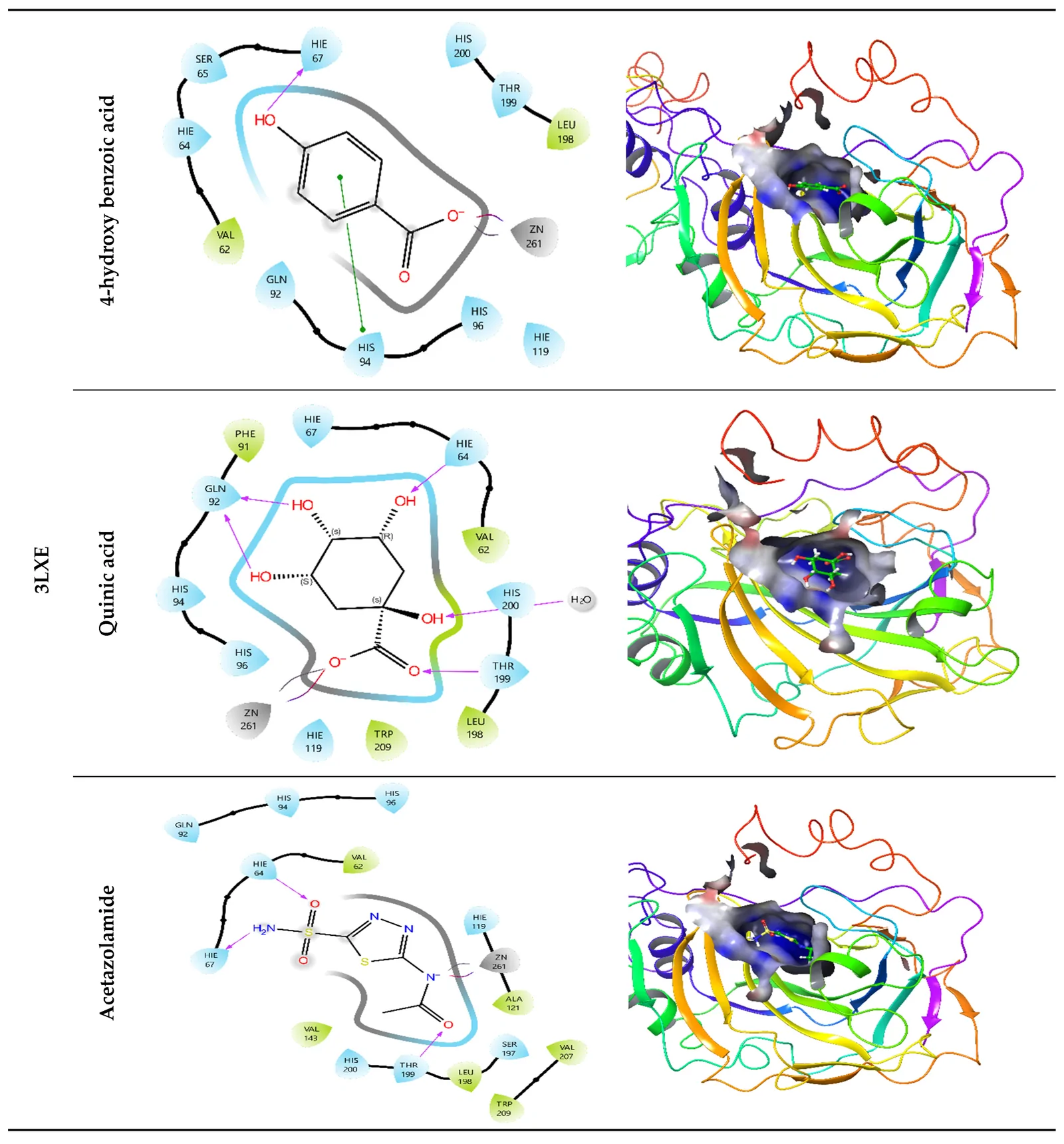

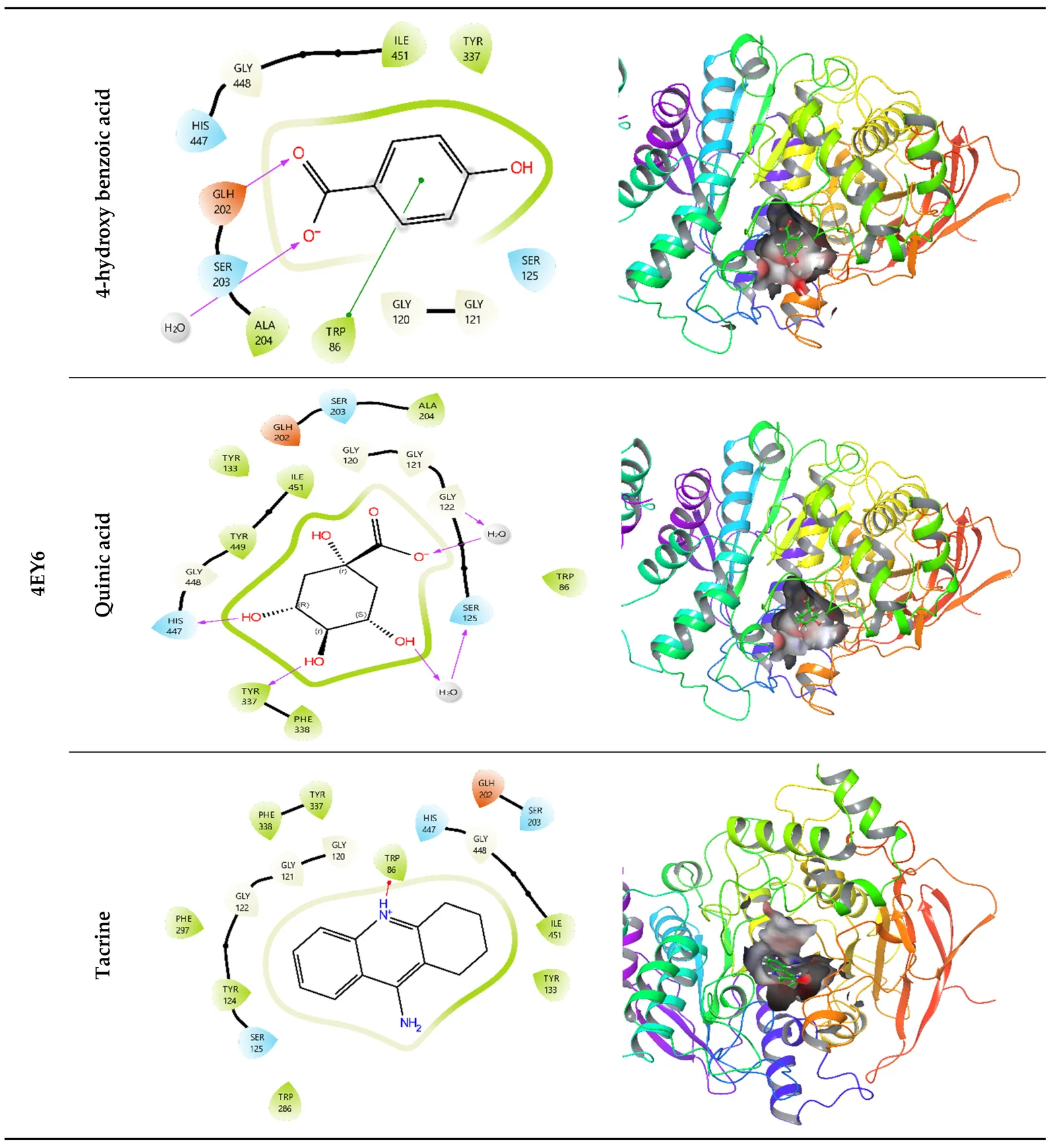

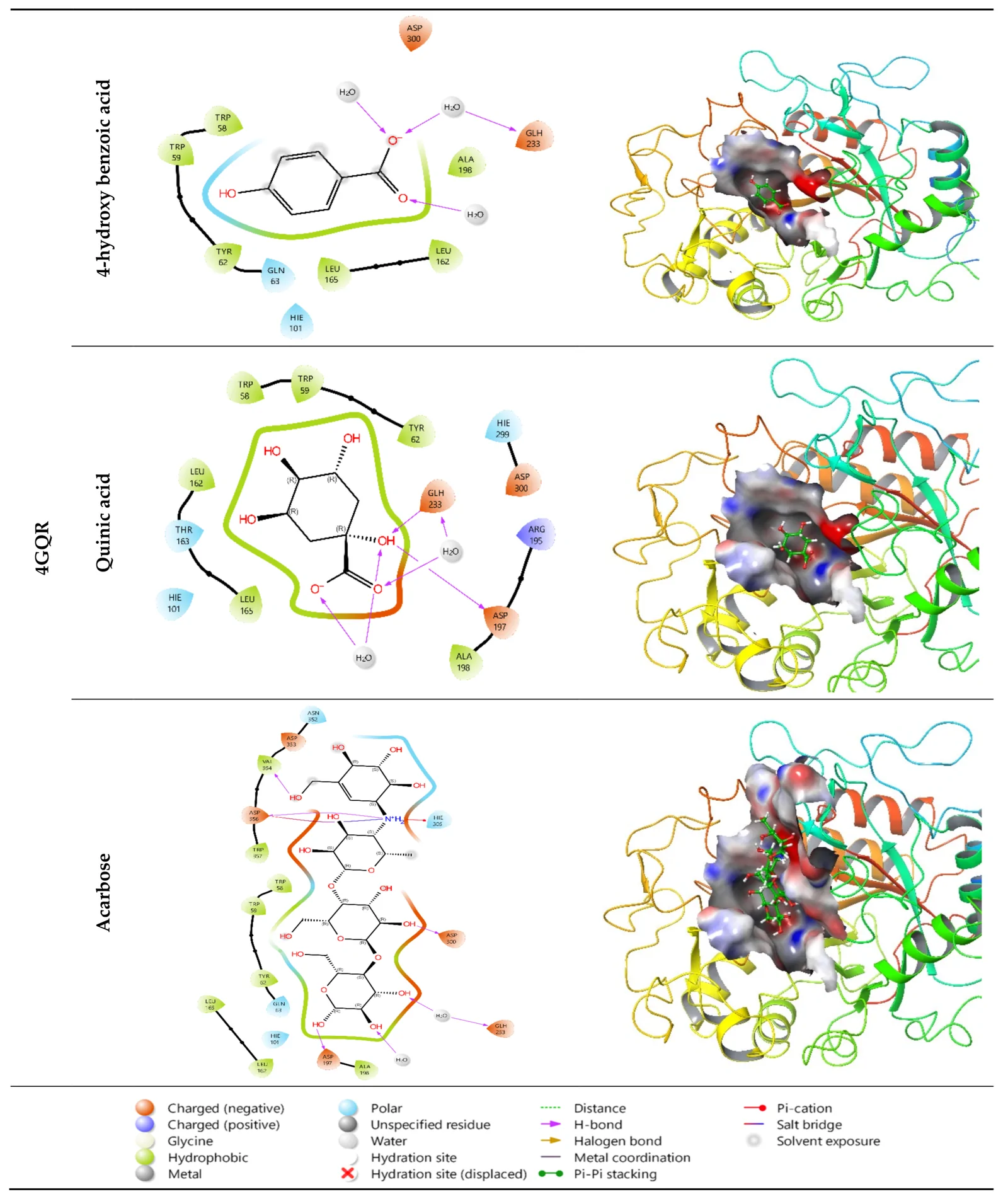
Two-dimensional (2D) and three-dimensional (3D) interaction profiles of 4-hydroxybenzoic acid, quinic acid, acetazolamide, tacrine, and acarbose with their respective target proteins.

The two-dimensional (2D) and three-dimensional (3D) interactions of the ligands 4-hydroxybenzoic acid, quinic acid, acetazolamide, tacrine, and acarbose with their target proteins are presented in Figure 4.

In this study, the interaction profiles of quinic acid with different target proteins (1P0I, 2QMJ, 3LXE, 4GQR, and 4EY6) were comparatively analyzed, revealing how the ligand’s highly hydroxylated, high-polarity structure adapts to different active site architectures. The docking results obtained indicate that the binding behavior of quinic acid is strongly dependent on the functional residue composition of the target protein.

In human butyrylcholinesterase (1P0I), quinic acid formed strong hydrogen bonding and electrostatic interactions with Glu238, which played a key role in ligand orientation. Additional stabilization was provided by water-mediated hydrogen bonds involving Arg242 and direct hydrogen bonding with Tyr282, suggesting that the relatively wide and hydrophilic active site of BChE favors binding of highly polar ligands.

In the maltase–glucoamylase structure (2QMJ), quinic acid interacted mainly with Asp443 and His600, supported by water-bridged hydrogen bonds that contributed to complex stability. The strong polar interactions with acidic residues such as Asp443 and Asp542 are consistent with the catalytic role of Asp/Glu residues in α-glucosidases and with previous reports describing quinic acid as a weak α-glucosidase inhibitor [57,58]. Similarly, in human carbonic anhydrase I (3LXE), quinic acid established hydrogen bonds with Gln92, Hie64, and the catalytically important Thr199, while additional stabilization was provided by hydrophobic contacts with residues such as Val62, Phe91, Leu198, and Trp209. The interaction with Thr199 agrees with the well-established binding patterns reported for carbonic anhydrase inhibitors [55,56].

Overall, these results indicate that quinic acid exhibits a weak to moderate binding profile characterized by hydrogen bonding, electrostatic complementarity, and water-mediated interactions. Such a binding pattern is typical of highly polar, naturally occurring polyhydroxylated compounds and supports the view that quinic acid may act as a multi-target, low-affinity modulator rather than a strong, single-target inhibitor. Quinic acid showed stable interactions within the active sites of several target proteins, including 4EY6 and 4GQR. In 4EY6, the hydroxyl groups of the ligand formed hydrogen bonds with His447 and Tyr337, while additional stabilization was provided by water-mediated interactions involving Ser125 and Gly122. These interactions contributed to proper ligand orientation and stabilization within the binding pocket. In the 4GQR structure, the carboxylate group of quinic acid interacted strongly with catalytic residues such as Asp197 and Glu233, forming hydrogen bonding and electrostatic interactions, some of which were supported by water-mediated bridges. The acidic nature of the active site and the highly polar, polyhydroxylated structure of quinic acid resulted in favorable electrostatic complementarity.

In general, docking analyses performed on 1P0I, 2QMJ, 3LXE, 4EY6, and 4GQR indicate that quinic acid binds predominantly through hydrogen bonds, water-mediated interactions, and electrostatic contacts with polar or acidic residues. The binding profile suggests a weak to moderate inhibitory or modulatory effect, which is consistent with the behavior commonly reported for naturally occurring phenolic and carboxylic acid derivatives that act through multiple low-affinity interactions [59,60].

## 4. Discussion

Antioxidant and enzyme-inhibitory abilities are commonly investigated as indicators of the biological potential of natural products. Antioxidant activity may indicate the capacity of a sample to counteract reactive species under experimental conditions, while inhibition of enzymes such as acetylcholinesterase, butyrylcholinesterase, α-glucosidase, and α-amylase may provide preliminary information on their potential relevance to pathways associated with neurological and glucose-metabolism-related processes. Therefore, the combined evaluation of these activities can provide useful preliminary evidence of the biological properties of natural products. However, such in vitro findings should be considered exploratory and should not be interpreted as direct evidence of therapeutic or clinical effects [61,62,63,64,65,66].

LC–MS/MS analysis revealed a diverse secondary metabolite profile in royal jelly (RJ), including quinic acid, 4-hydroxybenzoic acid, *p*-coumaric acid, protocatechuic acid, chlorogenic acid, caffeic acid, naringenin, luteolin, kaempferol, apigenin, and amentoflavone. These compounds mainly belong to phenolic acids and flavonoids, which are widely recognized for their antioxidant and enzyme-modulating properties. Among the identified metabolites, quinic acid was the most abundant compound, reaching 142,868 µg analyte/g extract. Quinic acid is an important intermediate of the shikimate pathway and has frequently been reported in plant- and bee-derived matrices [19,21]. Its reported antioxidant, anti-inflammatory, and neuroprotective properties may be associated with its ability to participate in redox-related processes and modulate oxidative stress [67,68,69,70]. Therefore, the high abundance of quinic acid, together with the presence of other phenolic compounds, may contribute to the biological activity observed for RJ.

Other identified phenolic acids, including 4-hydroxybenzoic acid, protocatechuic acid, *p*-coumaric acid, chlorogenic acid, and caffeic acid, are also associated with antioxidant activity through hydrogen/electron donation, metal chelation, and inhibition of lipid oxidation [28,71]. In addition, chlorogenic and caffeic acids have been reported to modulate carbohydrate-hydrolyzing enzymes and glucose metabolism [72,73,74]. *p*-Coumaric acid has also demonstrated antioxidant, antimicrobial, and cholinesterase-inhibitory properties [75,76,77,78]. Flavonoids detected in RJ, particularly naringenin, luteolin, kaempferol, apigenin, and amentoflavone, further increase the chemical diversity of the extract. These compounds have been widely associated with antioxidant, anti-inflammatory, neuroprotective, and enzyme-modulating activities [79,80,81,82]. Thus, the observed in vitro biological activity of RJ may reflect the combined contribution of multiple constituents rather than the effect of a single metabolite. However, further studies using isolated compounds and appropriate in vivo models are required to determine the specific contributions and potential biological relevance of individual constituents.

Consistent with this phytochemical profile, RJ exhibited appreciable in vitro antioxidant activity in both radical-scavenging and reducing-power assays. The IC_50_ values for DPPH^•^, ABTS^•+^, and DMPD^•+^ scavenging were 29.33 ± 2.21, 29.20 ± 1.48, and 21.65 ± 0.96 μg/mL, respectively. The reducing-power assays also supported this activity, with absorbance values of 0.702 ± 0.021, 1.444 ± 0.033, and 0.964 ± 0.072 for Fe^3+^ reduction, CUPRAC, and FRAP, respectively. Differences among antioxidant assays are expected because each method is based on different reaction mechanisms and experimental conditions; therefore, the complementary use of several assays provides a more comprehensive evaluation of antioxidant capacity [83,84]. The observed activity may partly be related to the detected phenolic acids and flavonoids. Nevertheless, RJ also contains proteins, peptides, and fatty acids with potential antioxidant properties, indicating that its antioxidant capacity is likely associated with the combined contribution of several chemical classes [83,85,86]. The measured total phenolic and flavonoid contents (53.147 ± 2.224 μg GAE/g and 23.559 ± 1.845 μg QE/g, respectively) further support the contribution of phenolic constituents to the observed activity.

The broad antioxidant profile was accompanied by considerable in vitro inhibition of hCA I, hCA II, AChE, BChE, α-glucosidase, and α-amylase, with IC_50_ values ranging from 1.12 to 2.11 μg/mL. RJ exhibited lower IC_50_ values than the reference inhibitors for hCA I, hCA II, AChE, and BChE under the experimental conditions used. Phenolics and flavonoids have previously been shown to interact with cholinesterases, supporting the possibility that some of the LC–MS/MS-detected constituents contribute to the observed activity [87,88]. RJ also inhibited α-glucosidase and α-amylase, with IC_50_ values of 2.04 and 2.11 μg/mL, respectively, compared with 2.85 μg/mL for acarbose. Similar inhibitory effects of RJ against carbohydrate-hydrolyzing enzymes have recently been reported, although activity may vary according to harvest conditions and chemical composition [89]. Collectively, these findings suggest that the diverse phytochemical composition of RJ may contribute to its broad enzyme-targeted activity. However, these in vitro results should not be directly interpreted as evidence of therapeutic efficacy, and further kinetic and in vivo studies are required.

Molecular docking results provided additional insight into the possible molecular interactions underlying these activities. Quinic acid showed favorable predicted binding to several target proteins, including BChE (1P0I), maltase–glucoamylase (2QMJ and 4GQR), CA I (3LXE), and AChE (4EY6). Its docking score was −5.674 Kcal/mol for BChE, lower than that of tacrine (−4.236 Kcal/mol), while its score against AChE (−6.662 Kcal/mol) was comparable to tacrine (−6.497 Kcal/mol). These interactions were predominantly associated with hydrogen bonding and electrostatic contacts, consistent with previous observations that hydroxylated phenolic compounds can interact with cholinesterase active sites [87]. For CA I, quinic acid showed a favorable docking score of −9.003 Kcal/mol compared with −6.296 Kcal/mol for acetazolamide, with interactions involving residues such as His64, Gln92, and Thr199. The interaction with Thr199 is particularly noteworthy because this residue is located near the catalytic region of carbonic anhydrase [55,56]. Nevertheless, docking scores should not be interpreted as direct evidence of superior inhibitory potency, particularly for metalloenzymes such as carbonic anhydrase, where Zn^2+^ coordination plays an important role in inhibitor binding [56].

In the maltase–glucoamylase structures, quinic acid showed moderate binding scores of −6.219 Kcal/mol (2QMJ) and −6.943 Kcal/mol (4GQR), whereas acarbose exhibited stronger binding, with scores of −8.020 and −7.599 Kcal/mol, respectively. This difference is consistent with the highly functionalized structure of acarbose and its ability to establish extensive interactions within carbohydrate-binding regions. Overall, the docking results indicate that quinic acid can accommodate different enzyme active sites through hydrogen bonds, electrostatic contacts, and water-mediated interactions. These findings, together with its high abundance in RJ and the experimentally observed antioxidant and enzyme-inhibitory activities, support quinic acid as a potential contributor to the multifunctional bioactivity of RJ. However, the docking data should be regarded as predictive evidence of molecular interaction rather than definitive proof of inhibition. Further studies using purified compounds, enzyme kinetics, binding assays, and in vivo models are warranted to determine the specific contribution of quinic acid and other RJ constituents to the observed biological effects.

The observed enzyme-inhibitory activities may be partly related to the structural characteristics of the identified phenolic compounds [90]. The hydroxyl groups of quinic acid and 4-hydroxybenzoic acid can provide multiple hydrogen-bonding interactions with amino acid residues within enzyme binding sites, while their carboxyl groups may contribute additional polar and electrostatic interactions. In particular, the higher predicted binding affinity of quinic acid toward α-glucosidase-related targets and carbonic anhydrase I compared with 4-hydroxybenzoic acid may be associated with its greater number and spatial distribution of hydroxyl groups, which can facilitate multiple hydrogen-bond interactions. Similarly, the phenolic hydroxyl and carboxyl functionalities of 4-hydroxybenzoic acid may support its positioning within the active sites. However, these structural features should be considered as potential contributors to inhibition, since docking scores alone cannot establish the precise structure–activity relationship or confirm the contribution of individual functional groups. Further experimental studies using structurally related compounds are required to validate these observations.

The main limitation of this study is the use of a single royal jelly sample, which limits the generalizability of the findings. In addition, moisture, protein, and 10-HDA contents could not be determined because of the limited sample amount. Therefore, the findings should be interpreted within the scope of the biochemical and computational approaches employed in this study. Further studies involving a larger number of independent royal jelly samples and comprehensive physicochemical and biochemical analyses are warranted to validate and further characterize the observed activities.

## 5. Conclusions

This study provides preliminary evidence that royal jelly possesses antioxidant and enzyme-inhibitory potential under the experimental conditions investigated, supported by complementary in vitro and in silico findings. LC–MS/MS analysis confirmed that royal jelly contains a diverse range of phenolic acids and flavonoids, while spectrophotometric assays revealed considerable total phenolic and flavonoid contents, consistent with its strong antioxidant potential. The antioxidant activity observed across radical scavenging and reducing power assays indicates that RJ exerts its effects through multiple redox mechanisms, likely driven by the synergistic action of its polyphenolic constituents.

RJ also exhibited notable inhibitory activity against carbonic anhydrases (hCA I and hCA II), cholinesterases (AChE and BChE), and carbohydrate-hydrolyzing enzymes (α-glucosidase and α-amylase). These findings indicate that the investigated royal jelly sample possesses a broad spectrum of enzyme-inhibitory abilities under the experimental conditions employed and provide biochemical evidence for its interactions with the investigated enzyme targets. Molecular docking analyses further supported these results by showing that major components, particularly quinic acid, can bind within enzyme active sites through hydrogen bonding, electrostatic interactions, and water-mediated contacts, providing a structural basis for the observed inhibition.

In conclusion, the combined biochemical and molecular modeling results indicate that royal jelly possesses multi-target biological activity and may serve as a promising natural source for functional foods, nutraceutical development, and future pharmacological research.

## Figures and Tables

**Table 1:** LC-MS/MS results of royal jelly (µg analyte/g extract).

N.	Secondary Metabolites	R.T.	M.I. (*m*/*z*)	F.I.	r^2^	Royal Jelly
1	Quinic acid	3.0	190.8	93.0	0.996	142.868
2	Fumaric acid	3.9	115.2	40.9	0.995	-
3	Aconitic acid	4.0	172.8	129.0	0.971	-
4	Gallic acid	4.4	168.8	79.0	0.999	-
5	Epigallocatechin	6.7	304.8	219.0	0.998	-
6	Protocatechuic acid	6.8	152.8	108.0	0.957	1
7	Catechin	7.4	288.8	203.1	0.999	-
8	Gentisic acid	8.3	152.8	109.0	0.997	-
9	Chlorogenic acid	8.4	353.0	85.0	0.995	0.448
10	Protocatechuic aldehyde	8.5	137.2	92.0	0.996	-
11	Tannic acid	9.2	182.8	78.0	0.999	-
12	Epigallocatechin gallate	9.4	457.0	305.1	0.999	-
13	Cynarine	9.8	515.0	191.0	0.999	-
14	4-OH Benzoic acid	10.5	137,2	65.0	0.999	75.942
15	Epicatechin	11.6	289.0	203.0	0.996	-
16	Vanillic acid	11.8	166.8	108.0	0.999	-
17	Caffeic acid	12.1	179.0	134.0	0.999	0.479
18	Syringic acid	12.6	196.8	166.9	0.998	-
19	Vanillin	13.9	153.1	125.0	0.996	-
20	Syringic aldehyde	14.6	181.0	151.1	0.999	-
21	Daidzein	15.2	417.1	199.0	0.996	-
22	Epicatechin gallate	15.5	441.0	289.0	0.997	-
23	Piceid	17.2	391.0	135/106.9	0.999	-
24	*p*-Coumaric acid	17.8	163.0	93.0	0.999	4.21
25	Ferulic acid-D3-IS	18.8	196.2	152.1	N.A.	-
26	Ferulic acid	18.8	192.8	149.0	0.999	-
27	Sinapic acid	18.9	222.8	193.0	0.999	-
28	Coumarin	20.9	146.9	103.1	0.999	-
29	Salicylic acid	21.8	137.2	65.0	0.999	-
30	Cyranoside	23.7	447.0	284.0	0.997	-
31	Miquelianin	24.1	477.0	150.9	0.999	-
32	Rutin-D3-IS	25.5	612.2	304.1	N.A.	-
33	Rutin	25.6	608.9	301.0	0.999	-
34	Isoquercitrin	25.6	463.0	271.0	0.998	-
35	Hesperidin	25.8	611.2	449.0	0.999	-
36	*o*-Coumaric acid	26.1	162.8	93.0	0.999	-
37	Genistin	26.3	431.0	239.0	0.991	-
38	Rosmarinic acid	26.6	359.0	197.0	0.999	-
39	Ellagic acid	27.6	301.0	284.0	0.999	-
40	Cosmosiin	28.2	431.0	269.0	0.998	-
41	Quercitrin	29.8	447.0	301.0	0.999	-
42	Astragalin	30.4	447.0	255.0	0.999	-
43	Nicotiflorin	30.6	592.9	255.0/284.0	0.999	-
44	Fisetin	30.6	285.0	163.0	0.999	-
45	Daidzein	34.0	253.0	223.0	0.999	-
46	Quercetin-D3-IS	35.6	304.0	275.9	N.A.	-
47	Quercetin	35.7	301.0	272.9	0.999	-
48	Naringenin	35.9	270.9	119.0	0.999	0.152
49	Hesperetin	36.7	301.0	136.0/286.0	0.999	-
50	Luteolin	36.7	284.8	151.0/175.0	0.999	0.069
51	Genistein	36.9	269.0	135.0	0.999	-
52	Kaempferol	37.9	285.0	239.0	0.999	0.374
53	Apigenin	38.2	268.8	151.0/149.0	0.998	0.075
54	Amentoflavone	39.7	537.0	417.0	0.992	0.079
55	Chrysin	40.5	252.8	145.0/119.0	0.999	0.366
56	Acacetin	40.7	283.0	239.0	0.997	0.256

N.: numbers, R.T.: retention time, M.I. (*m*/*z*): molecular ions of the standard analytes (*m*/*z* ratio), -: not detected, NA: not applicable. IS: internal standard, D3: deuterium isotope 3. FI (*m*/*z*): fragment ions, and *r*^2^: coefficient of determination.

**Table 2:** The most prevalent phenolic compounds found in RJ and their chemical structures.

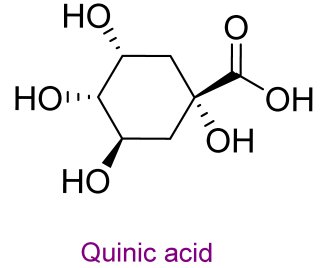	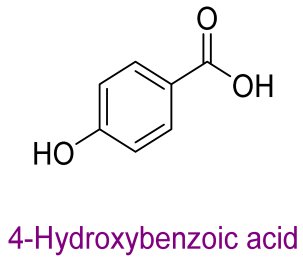	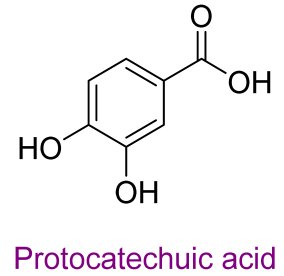
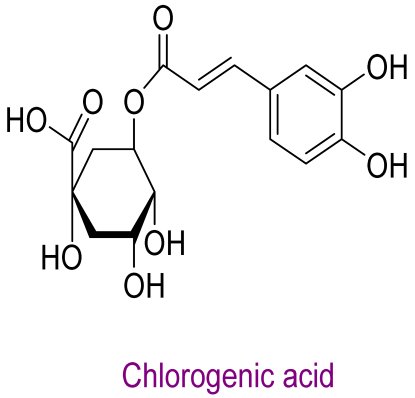		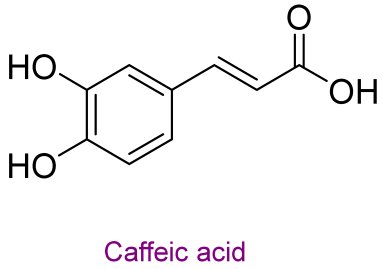
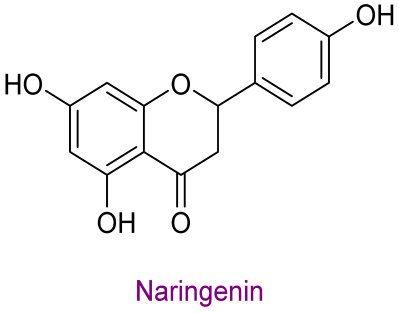	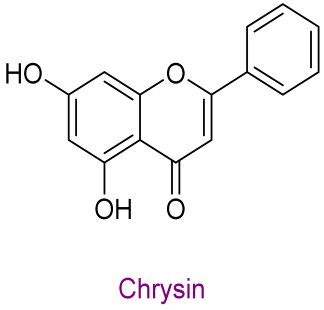	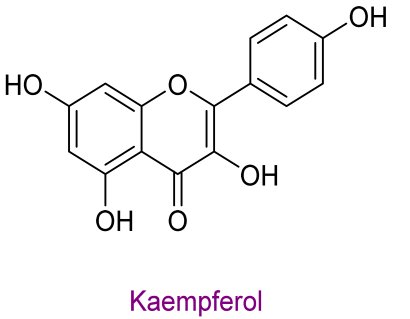
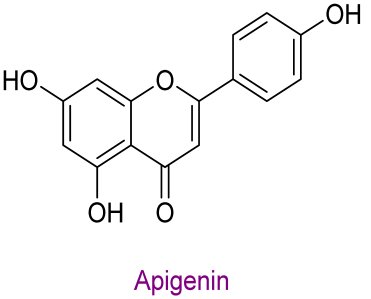	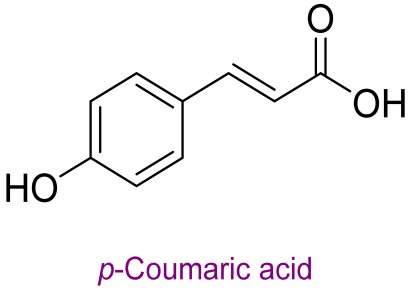	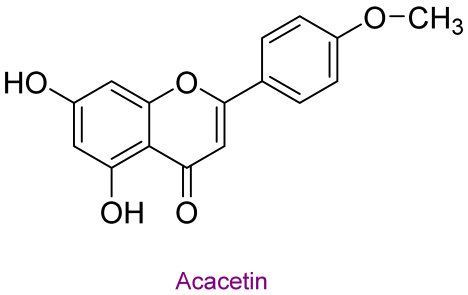
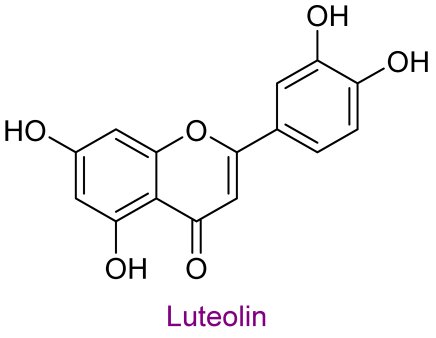	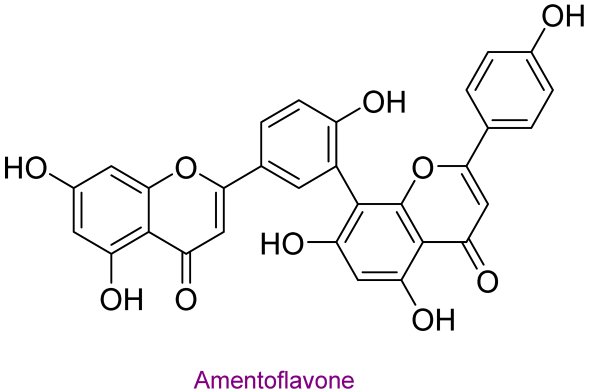	

**Table 3:** Half maximal inhibition concentration of RJ against DPPH, ABTS·^+^, and DMPD·^+^.

Sample and Standards	DPPH^·^ Scavenging		ABTS^·+^ Scavenging		DMPD^·+^ Scavenging	
	IC_50_ (μg/mL)	r^2^	IC_50_ (μg/mL)	r^2^	IC_50_ (μg/mL)	r^2^
Royal jelly	29.33 ± 2.21	0.9910	29.20 ± 1.48	0.9974	21.65 ± 0.95	0.9866
BHA	25.89 ± 1.23	0.9748	21.18 ± 1.03	0.9704	27.13 ± 1.08	0.9836
BHT	26.62 ± 3.21	0.9881	19.34 ± 1.02	0.9838	25.21 ± 1.03	0.9842
α-Tocopherol	25.62 ± 3.12	0.9501	33.61 ± 3.22	0.9854	29.63 ± 1.01	0.9938
Trolox	28.03 ± 1.02	0.9858	27.12 ± 0.85	0.9975	19.97 ± 0.96	0.9777
Ascorbic Acid	21.01 ± 2.03	0.9787	29.63 ± 2.05	0.9733	32.63 ± 1.03	0.9502

All values represent the meaning of three technical replicate measurements performed using the same independent sample and are presented as mean ± SD. The technical replicates were used to assess analytical repeatability and were not considered independent biological replicates; therefore, no statistical significance testing was performed. The IC_50_ concentrations are given as 60 µg/mL.

**Table 4:** Fe^3+^, CUPRAC, and FRAP reduction results of RJ.

Sample and Standards	Fe^3+^ Reduction Assay		CUPRAC Assay		FRAP Assay	
	λ_700_	r^2^	λ_450_	r^2^	λ_593_	r^2^
Royal jelly	0.702 ± 0.021	0.9902	1.444 ± 0.033	0.9854	0.964 ± 0.072	0.9763
BHA	1.771 ± 0.021	0.9862	2.102 ± 0.012	0.9852	0.984 ± 0.021	0.9785
BHT	1.256 ± 0.017	0.9933	1.865 ± 0.029	0.9921	1.012 ± 0.044	0.9869
α-Tocopherol	0.727 ± 0.011	0.9758	1.652 ± 0.029	0.9798	1.124 ± 0.020	0.9952
Trolox	0.797 ± 0.024	0.9684	1.744 ± 0.018	0.9951	0.992 ± 0.033	0.9850
Ascorbic acid	1.433 ± 0.013	0.9885	1.438 ± 0.022	0.9872	0.978 ± 0.029	0.9598

All values represent the meaning of three technical replicate measurements performed using the same independent sample and are presented as mean ± SD. The technical replicates were used to assess analytical repeatability and were not considered independent biological replicates; therefore, no statistical significance testing was performed. Concentrations are given as 60 µg/mL.

**Table 5:** Results of total phenolic and flavonoid content of royal jelly.

Total phenolics (µg GAE/g)	53.147 ± 2.224
Total flavonoid contents (µg QE/g)	23.559 ± 1.845

The values given are the three parallel measurements’ means ± SD (the term “triplicates” refers to three technical replicates of the same sample/extract, not three independent biological samples). GAE: gallic acid equivalents, QE: quercetin equivalents.

**Table 6:** hCA I, hCA II, AChE, BChE, α-glucosidase, and α-amylase enzyme inhibition results of royal jelly.

Bee Products	hCA I		hCA II		AChE		BChE		α-Glucosidase		α-Amylase	
	IC_50_	r^2^	IC_50_	r^2^	IC_50_	r^2^	IC_50_	r^2^	IC_50_	r^2^	IC_50_	r^2^
Royal jelly	1.12	0.9842	1.33	0.9870	1.44	0.9822	1.52	0.9935	2.04	0.9954	2.11	0.9802
Acetazolamide	2.21	0.9912	2.75	0.9787	-	-	-	-	-	-	-	-
Tacrine	-	-	-	-	2.38	0.9963	2.38	0.9883	-	-	-	-
Acarbose	-	-	-	-	-	-	-	-	2.85	0.9790	2.85	0.9921

Concentrations are given as (μg/mL). The reference inhibitors used for the respective enzymes were acetazolamide for hCA I and hCA II, tacrine for AChE and BChE, and acarbose for α-glycosidase and α-amylase.

**Table 7:** Docking score and Glide Emodel values (Kcal/mol) of 4-hydroxybenzoic acid, quinic acid, acetazolamide, tacrine, and acarbose against the selected target proteins obtained from molecular docking analyses.

Enzyme Target (PDB ID)	Ligand	Docking Score (Kcal/mol)	E Glide Model
Butyrylcholinesterase (BChE, E.C.3.1.1.8) (1P0I)	4-Hydroxy benzoic acid	−4.892	−23.626
	Quinic acid	−5.674	−36.535
	Tacrine	−4.236	−27.702
Maltase-glucoamylase (E.C.3.2.1.20) (2QMJ)	4-Hydroxy benzoic acid	−5.146	−25.926
	Quinic acid	−6.219	−46.123
	Acarbose	−8.020	−106.326
Carbonic anhydrase I (hCA I, E.C.4.2.1.1) (3LXE)	4-Hydroxy benzoic acid	−8.447	−65.110
	Quinic acid	−9.003	−78.557
	Azetazolamide	−6.296	−75.020
Acetylcholinesterase (AChE, E.C.3.1.1.7) (4EY6)	4-Hydroxy benzoic acid	−5.883	−30.298
	Quinic acid	−6.662	−40.577
	Tacrine	−6.497	−32.985
Maltase-glucoamylase (E.C.3.2.1.20) (4GQR)	4-Hydroxy benzoic acid	−6.604	−40.628
	Quinic acid	−6.943	−43.810
	Acarbose	−7.599	−105.501

## Data Availability

All relevant data are included in the manuscript. Artificial intelligence has been used in grammar checking.

## Abbreviations

Abbreviation	Full term
ABTS	2,2′-Azino-bis(3-ethylbenzothiazoline-6-sulfonic acid)
AChE	Acetylcholinesterase
BChE	Butyrylcholinesterase
BHA	Butylated hydroxyanisole
BHT	Butylated hydroxytoluene
CA	Carbonic anhydrase
CUPRAC	Cupric ion reducing antioxidant capacity
DMPD	N,N-Dimethyl-p-phenylenediamine
DPPH	2,2-Diphenyl-1-picrylhydrazyl
ESI	Electrospray ionization
FRAP	Ferric reducing antioxidant power
GAE	Gallic acid equivalents
hCA I	Human carbonic anhydrase I
hCA II	Human carbonic anhydrase II
IC_50_	Half-maximal inhibitory concentration
LC-MS/MS	Liquid chromatography-tandem mass spectrometry
LOD	Limit of detection
LOQ	Limit of quantification
MS/MS	Tandem mass spectrometry
p-NPG	p-Nitrophenyl-α-D-glucopyranoside
PNPA	p-Nitrophenyl acetate
QE	Quercetin equivalents
RJ	Royal jelly
R^2^	Coefficient of determination
TFC	Total flavonoid content
TPC	Total phenolic content
TE	Trolox equivalents
TE/g DW	Trolox equivalents per gram dry weight
TPTZ	2,4,6-Tris(2-pyridyl)-s-triazine
UHPLC	Ultra-high-performance liquid chromatography
